# Sphingosine 1-Phosphate Receptor 1 Signaling in Mammalian Cells

**DOI:** 10.3390/molecules22030344

**Published:** 2017-02-23

**Authors:** Nigel J. Pyne, Susan Pyne

**Affiliations:** Strathclyde Institute of Pharmacy and Biomedical Sciences, University of Strathclyde, Glasgow G4 0RE, UK; susan.pyne@strath.ac.uk

**Keywords:** sphingosine 1-phosphate, sphingosine 1-phosphate receptor 1, G-protein coupled receptor megaplex, receptor tyrosine kinase, immune trafficking, neovascularisation, cardiovascular, cancer

## Abstract

The bioactive lipid, sphingosine 1-phosphate (S1P) binds to a family of G protein-coupled receptors, termed S1P_1_-S1P_5_. These receptors function in, for example, the cardiovascular system to regulate vascular barrier integrity and tone, the nervous system to regulate neuronal differentiation, myelination and oligodendrocyte/glial cell survival and the immune system to regulate T- and B-cell subsets and trafficking. S1P receptors also participate in the pathophysiology of autoimmunity, inflammatory disease, cancer, neurodegeneration and others. In this review, we describe how S1P_1_ can form a complex with G-protein and β-arrestin, which function together to regulate effector pathways. We also discuss the role of the S1P_1_-Platelet derived growth factor receptor β functional complex (which deploys G-protein/β-arrestin and receptor tyrosine kinase signaling) in regulating cell migration. Possible mechanisms by which different S1P-chaperones, such as Apolipoprotein M-High-Density Lipoprotein induce biological programmes in cells are also described. Finally, the role of S1P_1_ in health and disease and as a target for clinical intervention is appraised.

## 1. Introduction

The bioactive lipid, sphingosine 1-phosphate (S1P), is produced by phosphorylation of sphingosine, catalysed by two isoforms of sphingosine kinase (SK1 and SK2), whereas its degradation involves cleavage by S1P lyase to produce (*E*)-2-hexadecenal and phosphoethanolamine [1]. S1P is also reversibly dephosphorylated by S1P phosphatase to regenerate sphingosine, the levels of which are additionally controlled by flux through the de novo ceramide synthesis and sphingosine salvage pathways. S1P in the plasma is produced by erythrocytes, vascular endothelial cells and, to a lesser extent, platelets (which lack S1P lyase). Release of S1P from activated platelets requires calcium-dependent and ATP (adenosine triphosphate)-dependent transporters whereas S1P is constitutively released in an ATP-dependent manner from erythrocytes, likely involving an ATP-binding cassette (ABC) type transporter [2]. The Spinster homologue 2 (Spns2) transporter passively exports S1P from vascular endothelial cells [3] (reviewed in [4]) and *Spns2* knockout mice are protected from airway inflammation, colitis, arthritis and experimental autoimmune encephalopathy (EAE) [5]. Thus, inhibitors of Spns2 may be usefully exploited to treat inflammatory diseases. Moreover, knockout of lymphatic endothelial *Spns2* reduces pulmonary metastasis via a mechanism that involves induction of a lymphopenia and an increase in effector T cell and natural killer (NK) cell number to enhance tumour cell killing in the lung [6]. 

S1P released from cells functions to stimulate a family of G protein-coupled receptors (GPCR), the S1P receptors (S1P_1_–S1P_5_) [7,8] on neighboring cells to induce biological responses such as growth, differentiation, cell migration and trafficking [1]. A major advance in understanding ligand binding to S1P receptors was the resolution of the atomic structure of S1P_1_, in complex with an antagonist. The authors proposed that lateral movement of S1P within the plane of the lipid bilayer and between two transmembrane helices is used to access the binding pocket of the receptor [9]. This suggests that released S1P partitions into the plasma-membrane to access S1P_1_. In this review, we present evidence that S1P_1_ can form complexes with G-protein and β-arrestin, which function together to regulate the extracellular signal regulated kinase-1/2 (ERK-1/2) pathway and cell migration. In addition, we describe the functioning of S1P_1_–Platelet derived growth factor receptor β (PDGFRβ) complexes and Apolipoprotein M-High-Density Lipoprotein-S1P (ApoM-HDL-S1P). We also review the role of S1P_1_ in health and disease and appraise the recent advances in clinical targeting of this receptor in disease.

## 2. Stable β-Arrestin-G-Protein-G Protein-Coupled Receptor Megaplexes

Before discussing S1P_1_ it is necessary to reflect on recent developments concerning the role of G-proteins and β-arrestins in GPCR signaling and desensitisation. Desensitisation of GPCRs involves the GRK2 catalysed phosphorylation of the GPCR and recruitment of β-arrestin, which overlaps the binding site for G-protein and thereby prevents functional coupling of the GPCR with G-protein [10]. β-Arrestins bind to the phosphorylated C-terminal tail and the transmembrane core of the GPCR and, in the latter case, this sterically impedes binding of G-protein. Therefore desensitised receptors cannot co-exist with G-protein and β-arrestin bound to the receptor at the same time. β-Arrestin also engages clathrin and adaptin 2 (AP-2) and facilitates endocytosis of GPCRs which can propagate signals to regulate, for instance, the ERK-1/2 pathway [11,12]. The strength and stability of the interaction between GPCR and β-arrestin has an effect on the kinetics of endocytosis [13]. The type of GPCR interaction with β-arrestin is designated as either class A or class B. This is a different classification from that used to define rhodopsin like (class A), secretin (class B) and metabotropic (class C) receptors. Class A receptors (e.g., β2-adrenergic receptor (β2AR)) exhibit weak β-arrestin–GPCR interactions and transient internalisation (which contributes to GPCR desensitisation) with rapid recycling. Class B receptors exhibit strong β-arrestin–GPCR interactions (e.g., V_2_ vasopressin receptor, V2R) and show sustained internalisation and signaling [13]. This can be facilitated by β-arrestin, which is a functional adaptor for several signaling molecules [14]. β-Arrestin dissociates from the class A receptor soon after internalisation, while remaining associated with class B receptors in endosomes. In addition, recent evidence has demonstrated that class B GPCRs induce sustained G-protein signaling in endosomes rather than being desensitised [15,16,17,18,19], suggesting that G-protein and β-arrestin are bound simultaneously with class B receptors.

Recent X-ray crystallographic analysis of the β2AR bound with G_s_ indicates that the N-terminal and C-terminal domains of the Gαs subunit interact with intracellular loop 2, transmembrane domain 5 (TM5), and TM6 of the β2AR [20]. Negative-stain electron microscopy (EM) using a β2 adrenergic-vasopressin 2 receptor chimera (β2V2R) in which the C-terminal tail of β2AR is exchanged for the C-terminal tail of V2R demonstrated that β-arrestin adopts two different binding modes [21]. First, β-arrestin can bind only to the phosphorylated receptor C-terminal tail (‘tail’ conformation) or second, can engage with the receptor in which, in addition to the tail interaction, a flexible loop in β-arrestin can insert into the transmembrane core of the receptor (‘core’ conformation). The binding of β-arrestin to the C-terminal tail only in class B GPCRs would therefore allow simultaneous engagement of G-protein with the receptor. Indeed, using single particle EM and biophysical measurements it has recently been shown that the β2V2R chimeric receptor can simultaneously interact with both G protein and β-arrestin in ‘so-called’ megaplexes which are present in endosomes and exhibit sustained G-protein signaling [22]. Therefore, the exchange of the C-terminal tail of the V2R converts the β2AR from a prototypical class A receptor to a class B receptor. Class B receptors contain clusters of Ser and Thr residues (phosphorylation sites) in the C-terminal tail that enable strong interaction with β-arrestin [13]. Therefore, Thomsen et al [22], proposed that the ‘tail’ conformation predominates for class B receptors and can therefore allow simultaneous binding of G-protein and β-arrestin in endosomes. These workers also suggested that as class A receptors lack these Ser/Thr clusters in the C-terminal tail then weak binding of β-arrestin in the ‘core’ conformation (with limited C-terminal tail interaction) predominates and prevents G-protein interaction. In this regard, class A GPCRs can use G-protein or β-arrestin to separately regulate the ERK-1/2 pathway [23]. These receptors will therefore, exist either in a GPCR-G-protein or GPCR–β-arrestin conformation. Thus, β-arrestin-biased agonists stabilise the GPCR–β-arrestin conformation and have inverse agonist activity on the GPCR–G-protein conformation as these receptor conformations exist in equilibrium. For example, the β-arrestin-biased angiotensin 1 receptor (AT1R) agonist Sar^1^,Ile^4^,Ile^8^ angiotensin II (SII) [24] stimulates ERK-1/2 activation and salt intake in the brain but blocks G protein-stimulated inositol phosphate formation and water intake [25].

It is possible that class B GPCRs might use both β-arrestin and G-protein βγ (Gβγ) sub-units together in an integrated manner to activate effector pathways. Indeed, β-arrestin 1 is positioned adjacent to the Gβγ subunits and several class averages indicate a direct interface between these proteins [22]. Moreover, biochemical analysis using the non-hydrolysable mimetic of guanosine triphosphate, guanosine diphosphate-tetrafluoroaluminate to dissociate Gα from Gβγ promotes interaction between β-arrestin 1 and Gβγ [22]. Therefore, it is possible that the association of Gβγ with β-arrestin might enable these proteins to function together in an integrated manner. Indeed, we demonstrated some years ago that S1P_1_ uses Gβγ and β-arrestin as multipliers of signal output to regulate ERK-1/2 in HEK 293 cells and airway smooth muscle cells [26,27] and this likely requires both G-protein and β-arrestin to be accommodated on S1P_1_ at the same time.

## 3. Sphingosine 1-Phosphate Receptor 1 Signaling

**Figure 1:**
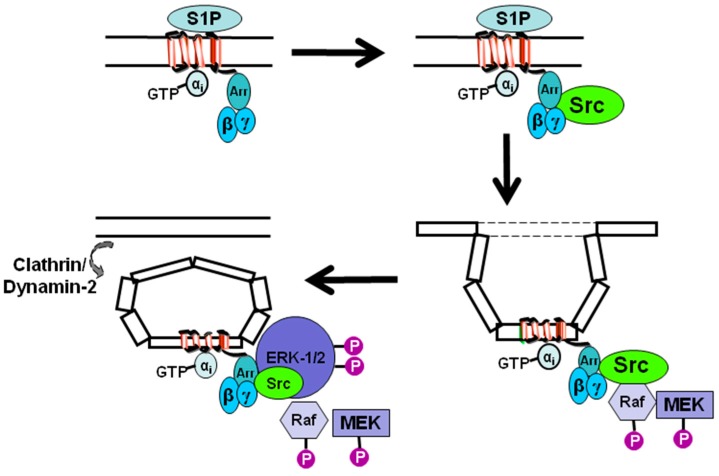
Schematic demonstrating the role of inhibitory G-protein (G_i_) and β-arrestin in regulating sphingosine 1-phosphate receptor 1 (S1P_1_) signaling in mammalian cells. β-Arrestin (Arr) associates with S1P_1_ and recruits c-Src to the receptor in response to S1P ligation of the receptor. G-protein βγ subunits are essential for subsequent activation of c-Src, Raf, MEK (mitogen activated protein kinase kinase) and ERK-1/2 (extracellular signal regulated kinase-1/2). S1P_1_ is internalised in endosomes via a β-arrestin-dependent mechanism and extracellular signal regulated kinase-1/2 (ERK-1/2) is recruited to the complex.

Evidence that S1P_1_ uses inhibitory G-protein (G_i_) and β-arrestin together to regulate the ERK-1/2 pathway (Figure 1) was based on the demonstration that pertussis toxin (which uncouples S1P_1_ from G_i_) or G-protein regulated kinase 2-ct (GRK2-ct, which sequesters Gβγ sub-units) or the clathrin binding domain of β-arrestin (which acts as a dominant negative by competing with endogenous β-arrestin for binding to clathrin) each inhibit S1P stimulation of ERK-1/2 activation by >90% in HEK 293 cells [26]. Moreover, S1P promotes formation of endosomes that contain S1P_1_ [27] and β-arrestin [28] and stimulates β-arrestin association with S1P_1_ in HEK 293 cells [29]. Further evidence to support the possibility that S1P_1_ can exhibit persistent signaling in endosomes is exemplified by the S1P_1_ modulator, FTY720 (2-amino-2-[2-(4-octylphenyl)ethyl]propane-1,3-diol) [19] which is active in its phosphorylated form. 

**Figure 2:**
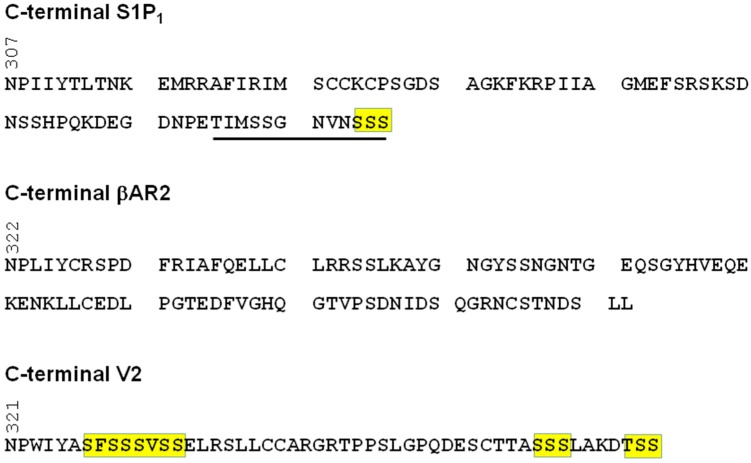
S1P_1_ C-terminal tail showing a Ser cluster at the extreme C-terminus possibly required for stable interaction with β-arrestin. The sequence underlined in the S1P_1_ C-terminal tail is essential for endocytosis of S1P_1_. Comparison is made with the class A β2 adrenergic receptor (β2AR) (that lacks Ser/Thr clusters) and the class B vasopressin receptor 2 (V2R) (which contains Ser/Thr clusters).

Clusters of Ser/Thr (e.g., SSS, SXSS, SSXS) in the C-terminal tail of GPCRs (which serve as phosphorylation acceptor sites) represent a barcode for formation of stable complexes between GPCR and β-arrestin [13]. In this regard, S1P_1_ contains an SSS motif at the extreme C-terminus (Figure 2). Furthermore, deletion of 12 amino-acids at the C-terminal tail reduces the S1P-stimulated phosphorylation of S1P_1_ and abolishes endocytosis of the receptor [30]. These findings identify the last 12 amino acids in the C-terminal tail of S1P_1_ as being critical for endocytosis and suggest that the SSS motif might represent a key β-arrestin interaction site. However, there is also evidence that stable GPCR–β-arrestin complexes can be formed without requirement for phosphorylation in the C-terminal tail. For instance, phosphorylation deficient receptor mutants for substance P [31], lutropin [32] and leukotriene B4 [33] still interact to form stable complexes with β-arrestin. 

A key signal tranducer of S1P_1_ is c-Src, which is recruited in response to S1P [26] and can receive stimulatory signals from Gβγ and β-arrestin, with the latter acting as an adapter for c-Src. S1P also stimulates a pertussis toxin-sensitive accumulation of dynamin-2 in lamellipodia in airway smooth muscle cells expressing S1P_1_ [26] (Figure 1). In addition, β-arrestin is a clathrin adaptor [12], which functions to load endosomes with GPCR. This provides an alternative mechanism by which β-arrestin can function as a multiplier of signal output by virtue of its ability to regulate the formation of endosomes containing competent S1P_1_ that can stimulate the ERK-1/2 pathway by a G-protein-dependent mechanism. 

The role of β-arrestin/dynamin-2 in S1P_1_ signaling has recently been confirmed by others. Reeves et al. used spinning disc confocal fluorescence microscopy and flow cytometry to demonstrate that S1P_1_ function was inhibited in cells depleted of β-arrestin 1/β-arrestin 2 or clathrin or AP-2 or by treating cells with the dynamin-2 inhibitor, dynasore-OH [34]. In addition, dynamin-2 was shown to be essential for low concentrations of S1P to induce S1P_1_ internalisation and continuous signaling in T cells, thereby regulating their egress from both thymus and lymph nodes [35]. In contrast, S1P was only capable of inducing a pulse of S1P_1_ signaling in T cells deficient in dynamin-2 and this was insufficient to promote T cell egress [35]. Thus, activated S1P_1_ uses a canonical route of clathrin- and dynamin-2-dependent endocytosis for persistent signaling.

We and others have used an S1P_1_ modulator, SB649146 to interrogate S1P_1_ signaling. We demonstrated that SB649146 is an inverse agonist (reduces constitutive basal S1P_1_-stimulated guanosine 5’-*O*-[gamma-thio]triphosphate binding), a competitive antagonist (with dihydrosphingosine 1-phosphate, a S1P_1_ specific agonist) and a partial agonist (weak stimulation of the ERK-1/2 pathway) of S1P_1_ [36]. SB649146 has no activity against S1P_2_, S1P_3_, lysophosphatidic acid receptor 1 or epidermal growth factor (EGF) and did not modulate the tyrosine phosphorylation of the PDGFRβ in response to PDGF [36]. Others have shown that SB649146 inhibits FTY720-phosphate stimulated ERK-1/2 activation in CCL39 lung fibroblasts over-expressing S1P_1_ [37] and reduces S1P-stimulated endothelial cell motility; and which is recapitulated by short interfering ribonucleic acid (siRNA) knockdown of S1P_1_ [38]. In addition, SB649164 reduced S1P-induced changes in transendothelial resistance without affecting intracellular S1P generated by the photolysis of caged S1P [39].

We have proposed that the S1P_1_ modulator SB649146 binds exclusively to and stabilises a low efficacy G_i_ coupling conformation of S1P_1_ and we demonstrated that SB649146 promotes endocytosis of S1P_1_ and β-arrestin, typical of stable interaction [28,36]. The poor stimulatory effect of SB649146 on G_i_ yields a low signal output resulting in a weak activation of ERK-1/2. It is possible that SB649146 induces the endocytosis of S1P_1_ (to a very much lesser extent than induced by S1P, possibly as G_i_ might also be involved in regulating loading of S1P_1_ in endocytic vesicles) via a β-arrestin-dependent mechanism, but that G-protein activation is poor and therefore stimulation of ERK-1/2 is weak. One can consider SB649146 as inducing weak internalisation of compromised S1P_1_. We also proposed that S1P binds exclusively to a high efficacy G_i_ and β-arrestin coupling conformation of S1P_1_, thus stabilising it and inducing a strong activation of ERK-1/2 [28,36]. Indeed, while we have demonstrated that SB649146 is a weak agonist for ERK-1/2 activation, it can competitively antagonise the S1P-stimulated activation of ERK-1/2 (by altering the equilibrium transition of each respective receptor conformation that can specifically bind S1P or SB649146 by mass action) [28,36]. Therefore, SB649146 might be a bona fide pharmacological modulator of stable GPCR–G-protein–β-arrestin complexes. Such pharmacological agents could function to ‘dial up’ or ‘dial down’ respective amounts of active G-protein and β-arrestin to determine signal output.

## 4. Sphingosine 1-Phosphate Receptor 1 Tyrosine Kinase Signaling Complexes

We have also shown that S1P_1_ forms a functional complex with the PDGFRβ in airway smooth muscle cells [26,27] and mouse embryonic fibroblasts [40] and HEK 293 cells [26,29]. Some years ago, we reported that the S1P_1_-PDGFRβ complex contains constitutively active S1P_1_ and uses G_i_, β-arrestin and PDGFRβ tyrosine kinase activity as multipliers of signal output in response to PDGF [26,28,36]. This promotes recruitment and activation of c-Src and stimulates the tyrosine phosphorylation of growth factor receptor-bound protein 2 associated binding protein 1 (Gab1), which can form a complex with growth factor receptor-bound protein 2 (Grb-2)-dynamn-2 [26]. The S1P_1_-PDGFRβ complex is then endocytosed via a β-arrestin/clathrin- and dynamin-2-dependent mechanism leading to activation of ERK-1/2 [26] and stimulation of cell migration [36,40]. Indeed, phosphorylated ERK-1/2 co-localises with the S1P_1_-PDGFRβ complex in endosomes in response to PDGF [26]. 

The S1P_1_ modulator SB649146 might also reduce the availability of Gβγ subunits for use by the PDGFRβ (which is in a complex with the high efficacy G_i_/β-arrestin coupling conformation of S1P_1_), thereby inhibiting PDGF-stimulated activation of ERK-1/2 and cell migration [28,36]. We have proposed that SB649146 binds to the low efficacy G_i_ coupling conformation of S1P_1_, (which does not bind PDGFRβ) and reduces the concentration of the high efficacy G_i_/β-arrestin S1P_1_ conformation associated with PDGFRβ by mass action [28,36]. We have demonstrated existence of the different conformational states of S1P_1_ using immunofluorescent staining in airway smooth muscle cells. Treatment of cells with PDGF stimulates the endocytosis of the S1P_1_-PDGFRβ complex [26,27,36]. This pool of S1P_1_ is likely to represent the high efficacy G_i_/β-arrestin coupling conformation. In contrast, SB649146 weakly stimulates the endocytosis of S1P_1_ and this occurs in the absence of PDGFRβ [36]. This second pool of S1P_1_ is likely to represent the low efficacy G_i_ coupling conformation [28,36]. In addition, SB649146 inhibits the PDGF-stimulated endocytosis of the S1P_1_-PDGFRβ complex and also reduces the S1P-stimulated endocytosis of S1P_1_ [36].

Interestingly, recent studies have shown that S1P_1_ can be regulated by tyrosine phosphorylation, thereby providing additional evidence of a role for receptor tyrosine kinase (RTK) or c-Src in the regulation of S1P_1_ function [41]. The Y143 site was shown to be required for S1P_1_ internalization in response to S1P and this was associated with defective endothelial barrier enhancement induced by S1P. Overexpression of phosphorylation deficient (Y143F) or phosphorylation mimicking (Y143D) mutants failed to internalise or exhibited very high receptor internalisation respectively [41]. Therefore, Y143 regulates cell surface expression of S1P_1_ and this is required for the endothelial barrier repair function of S1P.

There are other examples of S1P_1_ forming complexes with other RTK. For example, in follicular thyroid carcinoma ML-1 cells, the vascular endothelial growth factor receptor-2 (VEGFR-2) forms a complex with S1P_1_. The S1P_1_–VEGFR-2 complex interacts with ERK-1/2 and protein kinase Cα [42]. In addition, S1P treatment of mouse embryonic stem (ES) cells promotes β-arrestin binding to S1P_1/3_ and this leads to activation of c-Src [43]. This is associated with the stimulation of cell proliferation. S1P also increases the binding of S1P_1/3_ with VEGFR-2 and promotes VEGFR-2 phosphorylation, which was blocked by β-arrestin siRNA, and the c-Src inhibitor, PP2 [43]. There are other examples of GPCR–RTK complexes. For instance, insulin-like growth factor 1 (IGF-1) is associated with the constitutively active G_i_ coupled chemokine receptor type 4 (CXCR4). This complex promotes migration of MDA-MB-231 breast cancer cells [44]. Furthermore, constitutively active pituitary adenylate cyclase-activating peptide type 1 (PAC1) receptor associates with IGF-1R to regulate neuronal survival [45]. Interestingly, both CXCR4 and PAC1 are class B receptors based on the presence of serine clusters in the C-terminal tail required for stable β-arrestin interaction, and suggesting that along with S1P_1_, there might be specificity for class B receptors with RTKs.

Other S1P receptor sub-types, such as S1P_2_ deploy endosomal signaling [43]. In this case, phosphorylation of ezrin (of the ezrin-radixin-moesin family of adapter molecules, required for cancer cell invasion) in response to EGF requires SK2 and intracellular S1P_2_ and involves an intracrine action of intracellular S1P possibly made available by a close proximity localisation of Spns2 with S1P_2_ in endosomes [46].

## 5. Sphingosine 1-Phosphate Receptor 1 and Regulator of G-Protein Signaling 12

**Figure 3:**
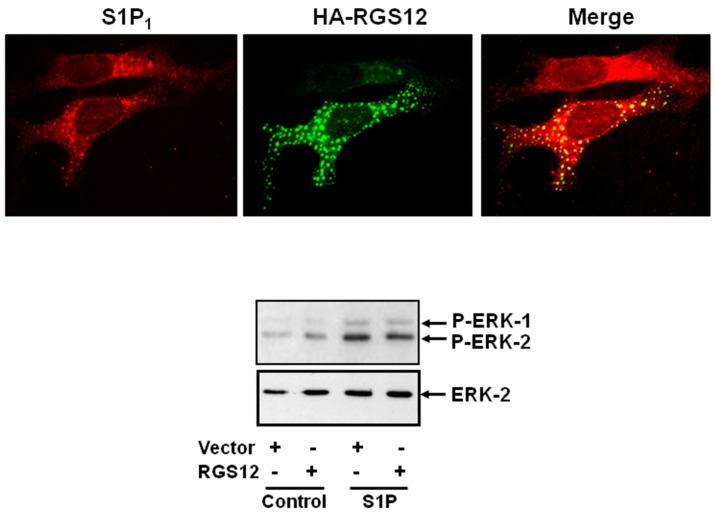
RGS-12 (Regulator of G-protein signaling-12) co-localises with the S1P_1_ in airway smooth muscle cells and reduces S1P-stimulation of the ERK-1/2 pathway. Airway smooth muscle cells were transfected with plasmid constructs encoding myc-tagged S1P_1_ and hemagglutinin (HA) tagged RGS12 and stimulated with S1P (1 μM, 5 min). The data shows that RGS12 co-localises with S1P_1_ in cytoplasmic vesicles. It remains to be determined whether S1P_1_ in these vesicles is competent to signal. However, RGS12 dampens the activation of ERK-1/2 by S1P in these cells. The results are representative of three independent experiments.

We have previously shown that Regulator of G-protein Signaling 12 (RGS12) modulates PDGFRβ signaling [47]. Firstly, over-expression of RGS12, RGS12 (Post synaptic density protein (PDZ)/Phosphotyrosine binding domain (PTB) N-terminus or RGS12 PTB domain decreased ERK-1/2 activation in response to PDGF in airway smooth muscle cells. Secondly, the RGS12 PDZ/PTB domain N-terminus and RGS12 PDZ domain associate with the PDGFRβ [47]. In addition recombinant RGS12 and the isolated PDZ/PTB domain N-terminus co-localise with PDGFRβ in cytoplasmic vesicles [47]. Similarly, we show here that S1P_1_ co-localises with recombinant RGS12 in these cytoplasmic vesicles and overexpression of RGS12 reduces S1P-stimulation of ERK-1/2 in airway smooth muscle cells (Figure 3).

## 6. Sphingosine 1-Phosphate Receptor 1 and Sphingosine 1-Phosphate Carriers/Chaperones

Plasma S1P is associated with carrier/chaperone proteins, such as albumin and high density lipoprotein (HDL). Christofferson et al. [48] were the first to show that HDL-S1P was bound to apolipoprotein M (ApoM). S1P binds to an amphiphilic pocket in the lipocalin fold of ApoM. From a functional perspective, ApoM-HDL stimulated endocytosis of S1P_1_ and promoted activation of ERK-1/2, protein kinase B (PKB), endothelial cell migration and formation of adherent junctions. These studies revealed that S1P in ApoM-HDL was protective towards endothelial function. Recent studies have demonstrated that S1P_1_ signaling in endothelial cells is more sustained in response to HDL-bound ApoM-S1P compared with albumin-bound S1P [49]. This might involve an HDL-bound S1P-S1P_1_ receptor/β-arrestin complex, resident at the plasma-membrane, which reduces tumour necrosis factor alpha (TNFα)-induced activation of nuclear factor kappa B (NF-κB) and intercellular adhesion molecule 1 (ICAM-1) expression. In contrast, albumin-bound S1P-S1P_1_ receptor is endocytosed and involves G_i_-mediated signaling [50]. Since it is well established that S1P_1_ uses a β-arrestin-dependent mechanism to regulate endocytosis of S1P_1_, we suggest that HDL-bound S1P-S1P_1_ might be specifically trapped at the plasma membrane with β-arrestin. This could be achieved if HDL-bound S1P-S1P_1_ is associated with an accessory protein that prevents endocytosis of S1P_1_ and therefore initiates a plasma-membrane S1P_1_ receptor/β-arrestin signaling programme which is anti-inflammatory.

ApoM-S1P is involved in regulating specific cell biology. For instance, ApoM-S1P is dispensable for lymphocyte trafficking but limits lymphopoiesis via S1P_1_ expressed on bone marrow lymphocyte progenitors [51]. Proliferation of *Lin*(−) *Sca-1*(+) *cKit*(+) haematopoietic progenitor cells (LSKs) and common lymphoid progenitors (CLPs) in bone marrow is increased in mice that are deficient in ApoM. Moreover, overexpression of S1P_1_ suppresses proliferation of LSK and CLP cells in vivo and decreases lymphopoiesis in vitro. The failure to deliver S1P in *Apom*^−/−^ mice to bone progenitors results in severe EAE. This is due to increased lymphocytes in the central nervous system (CNS) and breakdown of the blood-brain barrier [51]. In addition, activation of endothelial S1P_1_ by HDL-S1P induces liver regeneration and suppresses fibrosis. Indeed, in mice deficient in HDL-S1P, liver regeneration after partial hepatectomy is reduced and associated with aberrant vascular remodelling, thrombosis and peri-sinusoidal fibrosis [52]. 

Studies of S1P_1_ and its interaction with accessory proteins and/or co-receptors are being facilitated by in vivo reporters of S1P_1_ signaling. For instance, a green fluorescent protein (GFP) expression reporter following activation of a S1P_1_/transcription factor fusion protein that is cleaved by a β-arrestin/protease fusion protein has been developed [53]. This mouse was used to demonstrate that lipopolysaccharide (LPS)-mediated systemic inflammation leads to the activation of S1P_1_ in endothelial cells and hepatocytes in vivo [53]. Another model involves differential internalisation of a competent S1P_1_/GFP fusion protein compared with an S1P binding deficient S1P_1_:RFP fusion protein [54]. Therefore, these S1P_1_ reporter mice will also allow the tissue-specific interrogation of S1P_1_ activation in disease models.

## 7. Sphingosine 1-Phosphate Receptor 1 in Health and Disease

### 7.1. Sphingosine 1-Phosphate Receptor 1 and Immune Function

**Figure 4:**
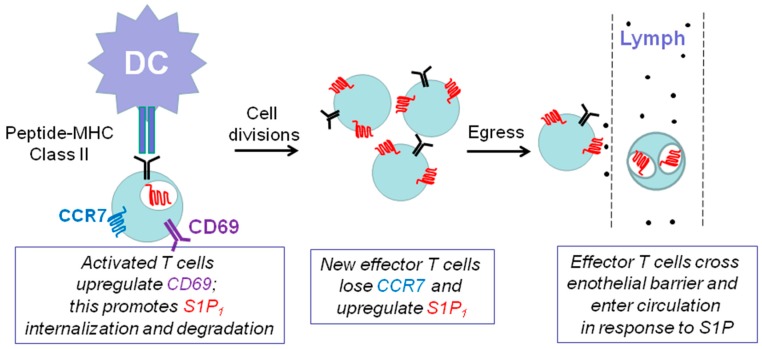
Schematic demonstrating the role of S1P/S1P_1_ in T-cell trafficking in the immune system. Engagement with Dendritic Cells (DC) presenting antigen in a major histocompatibility class II (MHC class II) complex causes expansion of CD4^+^ (Cluster of Differentiation), which requires retention in lymph nodes and is achieved by the chemokine receptor 7 (CCR7) and CD69-mediated down-regulation of S1P_1_. Newly formed effector T-cells then lose CCR7 and up-regulate S1P_1_ so that they can sense an S1P gradient between lymph nodes and lymph thereby allowing their egress into lymph.

The sphingosine-like molecule, FTY720 (or fingolimod, formulated as Gilenya^TM^) is used as the first oral treatment for relapsing and remitting multiple sclerosis [55]. SK2 catalyses the phosphorylation of FTY720 and the FTY720-phosphate is released from cells to stimulate S1P receptors. Chronic exposure to FTY720 (FTY720-phosphate) induces a decrease in S1P_1_ levels thereby reducing inflammatory T cell invasion of the CNS and reducing multiple sclerosis disease progression. FTY720 phosphate induces functional antagonism due to proteasomal degradation of S1P_1_ and this prevents the egress of T-cells as these are S1P_1_ null and are unable to respond to a critical S1P gradient between lymph and lymph nodes (Figure 4). Th17 cells found primarily within central memory T cells are reduced (including retinoid related orphan receptor γt (RORγt) and interleukin-17 (IL-17)-producing T cells) by >90% in response to FTY720 [56].

**Figure 5:**
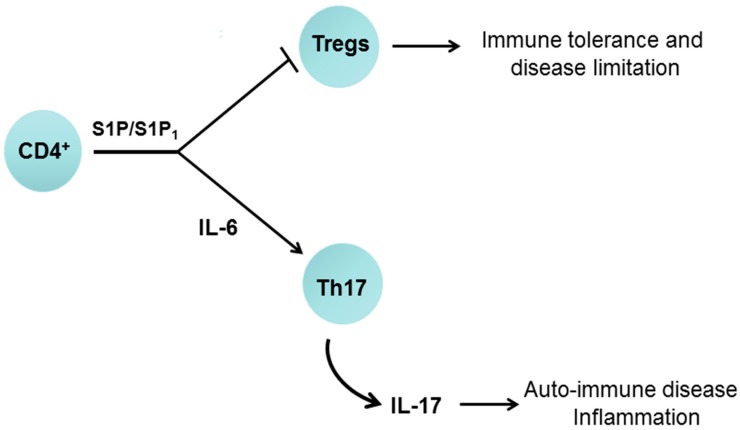
Schematic demonstrating the role of S1P/S1P_1_ in T-cell differentiation. S1P acting via S1P_1_ can promote an interleukin-6 (IL-6)-dependent polarisation of CD4^+^ cells to form T helper 17 (Th17) cells, which release IL-17. S1P binding to S1P_1_ also inhibits regulatory T cells (T(reg)) formation thereby exacerbating the polarisation of Th17 cells.

Multiple sclerosis involves an unrestrained autoimmune Th17 response. Indeed, S1P enhances Th17 cell polarisation [57] found primarily within central memory T cells. The mechanism is promoted by an S1P/S1P_1_-dependent increase in signal transducer and activator of transcription 3 (STAT3) and IL-6 formation; this S1P_1_ dependent pro-inflammatory pathway was first, demonstrated in cancer cells [58]. Moreover, STAT3 increases RORγt expression [59]. In addition, STAT3 knockout reduces Th17 polarisation [59] and STAT3 binds directly to the IL-17 promoter [60]. Significantly, knockout of RORγt prevents Th17 polarisation [61]. Therefore, S1P likely stimulates Th17 polarisation via a RORγt-dependent mechanism. S1P/S1P_1_ is also required for egress of IL-17-producing Vγ4+ γδ T cells from the lymph nodes under homeostatic and inflammatory conditions [62]. S1P/S1P_1_ also inhibits T(reg) function [63], thereby preventing the suppressive effect of T(reg) on Th17 formation (Figure 5). S1P/S1P_1_ blocks the differentiation of thymic T(reg) precursors and the function of mature T(reg) cells and therefore modulates T(reg) cell-mediated immune tolerance. This occurs through the S1P_1_-dependent activation of the PKB/mammalian target of rapamycin (mTOR) pathway, which regulates the adaptive immune response [63].

The resistance of certain relapsing and remitting multiple sclerosis patients to FTY720 might be due to polymorphism in the *S1P_1_* receptor gene. For instance, Ile45 to Thr and Gly305 to Cys mutations (found in patients) renders the S1P_1_ receptor resistant to FTY720-induced degradation [64]. In addition, phosphorylation on S351 in S1P_1_ has been identified as a critical regulator of receptor internalisation [65]. Indeed, mutant mice expressing phosphorylation-deficient receptors (S1P_1_(S5A)) develop severe EAE involving Th17 cells. S1P_1_ activates the Janus activated kinase (JAK)/STAT3 pathway via IL-6 thereby enhancing Th17 polarisation and worsening neuro-inflammation, which is likely to represent a key mechanism in multiple sclerosis [65]. These findings suggest that plasma-membrane S1P_1_ regulates Jak-STAT3/IL-6 as this is enhanced in cells where S1P_1_ is resistant to endocytosis.

S1P_1_ is required for the exit of mature B cells from secondary lymphoid organs. In addition, S1P_1_ deficiency reduces the number of newly generated immature B cells in the blood [66]. This is due to enhanced apoptosis of immature B cells in contact with the vascular compartment. Forced expression of CD69, a negative regulator of S1P_1_ receptor expression also reduced the number of immature B cells in the blood. Chemokine receptor (CCR7 and CXCR4) recycling and S1P_1_ are also implicated in chronic lymphocytic leukemia pathogenesis and clinical outcome [67].

### 7.2. Sphingosine 1-Phosphate Receptor 1 and the Nervous System

The S1P_1_ modulator, FTY720 also reduces astrogliosis and supports nerve remyelination and recovery [68]. Moreover, the S1P_1_-specific agonist (AUY954) which reduces EAE in SJL/J autoimmune susceptible mice induces a decrease in lymphocyte numbers in the CNS without interfering with trafficking of plasmacytoid dendritic cells (pDCs) to the CNS [69]. pDCs are important in limiting the autoimmune responses during EAE. S1P_1_ deficiency also delays differentiation of oligodendrocyte progenitors (OPCs) into oligodendroglial cells (OLGs); accompanied by decreased levels of myelin basic protein but not myelin-OLG glycoprotein [70]. S1P_1_-deficient OLGs exhibited slower process extension concomitant with reduced phosphorylated ERK-1/2 and p21-activated kinase (PAK) levels. Therefore, S1P_1_ regulates OLG development, morphological maturation and early myelination. FTY720 phosphate binding to S1P_1_ also reduces activated microglial production of pro-inflammatory mediators, TNFα, IL-1β and IL-6 and increases microglial production of brain-derived neurotrophic factor and glial cell-derived neurotrophic factor that are protective [71]. 

### 7.3. Sphingosine 1-Phosphate Receptor 1 and Neovascularisation

VEGF promotes sprouting of endothelial cells to produce capillary tubes that are then stabilised by S1P. In this regard, S1P binding to S1P_1_ inhibits VEGFR2 signaling and angiogenesis in endothelial cells [72,73] by promoting stabilisation of VE-cadherin at endothelial junctions [72]. In other words, VEGF starts the process of blood vessel formation and S1P finishes it. In wet age-related macular degeneration, atrophy of the retinal pigment epithelium (which is a proposed source of S1P, and which can undergo Epithelial Mesenchymal Transition (EMT) to become contractile myofibroblasts [74]) and/or development of new unstable blood vessels results in death of photoreceptors and loss of central vision. VEGF promotes sprouting of endothelial cells in the choroidal region behind the retina, which leak/hemorrhage. This can be considered as pathological angiogenesis, where blood vessels have proceeded to some degree of maturity but have not fully matured. Removal of VEGF prevents sprouting and branching and induces regression of these unstable vessels. As VEGF is a survival signal, its removal can induce apoptosis of endothelial cells to regress blood vessels. Intervention with anti-VEGF therapeutics such as Avastin [75] (a humanised antibody that binds VEGF) halts vessel sprouting, regresses immature blood vessels and elicits a small improvement in visual acuity [75]. It follows that targeting S1P_1_ could achieve the same therapeutic utility in terms of preventing full maturation and promoting vessel regression in response to anti-VEGF therapy. In support of this approach, anti-S1P monoclonal antibodies have been shown to markedly reduce choroidal neovascularisation lesion volume, sub-retinal fibrosis and pericyte recruitment in a murine model of laser-induced rupture of Bruch’s membrane [76]. However, in a first clinical phase IIa study (Nexus trial), anti-S1P antibody treatment did not meet its primary or key secondary end points.

### 7.4. Sphingosine 1-Phosphate Receptor 1 and the Heart

S1P mediates multiple pathophysiological effects in the cardiovascular system. For instance, cardiac S1P levels are increased post-myocardial infarction (MI) and this is associated with increased SK1 and S1P_1_ expression [77]. In addition, β1-adrenergic receptor stimulation of S1P/S1P_1_ underlies the pro-inflammatory response in cardiomyocytes [77]. Administration of FTY720, to functionally antagonise S1P_1_, reduces chronic cardiac inflammation, and improves cardiac remodeling and dysfunction in vivo post-MI [77]. Moreover, S1P_1_ is required for normal cardiac development. Thus, the conditional knockout of the S1P receptor 1 (*S1pr1*) results in ventricular non-compaction and ventricular septal defects leading to perinatal lethality [78].

The transition from beneficial hypertrophy (which enables the heart to tolerate high blood pressure) to heart failure is governed by the formation of new blood vessels that can reoxygenate the heart [79]. Angiogenesis can prevent the development of malfunctional hypertrophy and heart failure and this is regulated by the *p53* gene [80]. Thus, hypoxia has been shown to increase p53 expression via a hypoxia-inducible factor 1 alpha (HIF1α)-dependent mechanism and p53 inhibits angiogenesis by reducing HIF1α in a negative feedback manner [80]. Therefore, high p53 expression is a causative factor in the development of heart failure, a result of apoptosis of cardiomyocytes. Therapeutic approaches that increase angiogenesis in the heart have been suggested as a means to prevent the transition from beneficial hypertrophy to heart failure. However, approaches using angiogenic factors have largely failed because this results in formation of immature vessels that cannot be sustained. In this case, stimulation of S1P_1_ and recruitment of mural cells to enable full maturation of newly formed blood vessels is likely to normalise the heart vasculature and offer an improved efficacy with VEGF in preventing transition to heart failure. In addition, FTY720 has been employed to reduce cardiac remodelling post myocardial infarction in animal models [77]. Although this has been interpreted as being mediated through S1P_1_-dependent changes in inflammation [77], it is possible that the agonistic effect of FTY720 phosphate on S1P_1_ might mature blood vessels to protect against deleterious cardiac remodelling. 

### 7.5. Sphingosine 1-Phosphate Receptor 1 and Cancer

There is substantial evidence implicating S1P in cancer including SK1-induced transformation, EMT, invasiveness, regulation of cancer cell survival and replicative immortality, regulation of tumour neovascularisation and changes in metabolism [81]. In this regard, high expression of S1P_1_ in tumours from estrogen receptor positive (ER^+^) breast cancer patients is associated with shorter disease-specific survival [82]. S1P_1_ has also been shown to co-localise with SK1 and filamin A in lamellipodia in filamin A-expressing A7 melanoma cells, and this is required for cell motility and is blocked by an S1P_1_ antagonist [83]. S1P_1_ is also implicated in the neovascularisation of tumours. For instance, FTY720 inhibited primary and metastatic tumour growth in a mouse model of melanoma growth [84]. This was associated with inhibited tumour-associated angiogenesis and decreased tumour cell proliferation. Recent studies have demonstrated that EMT of hepatocellular carcinoma cells involves S1P/S1P_1_-dependent phosphoinositol-3-kinase (PI3K)/PKB activation and increased expression of metalloproteinase 7 (MMP7) and shedding and loss of syndecan-1 [85]. This loss of syndecan-1 promotes a TGFβ (Transforming growth factor β)-dependent EMT that is implicated in hepatocellular carcinoma metastasis [86]. S1P/S1P_1_ is also involved regulating the expression of hypoxia-inducible factor 2 alpha (HIF2α), which can drive aggressive cancer [87]. Thus, siRNA knockdown of S1P_1_ and Spns2 blocks HIF2α accumulation, suggesting that S1P might exert so-called ‘inside-out’ signaling, where SK1 catalyses formation of S1P, which is released from cells to act on S1P_1_ in an autocrine manner to regulate cancer cell growth. Similarly, estrogen (E2) activates SK1 and promotes internalisation of S1P_1_; the latter is required for activation of PKB/endothelial nitric oxide synthase (eNOS) in endothelial cells and which regulates endothelial cell migration and tube formation [87]. Therefore endothelial cell S1P_1_ might function as a nodal point in E2 signaling and play an important role in neovascularisation of estrogen-dependent tumours. SK1 expression is also increased in invasive cancer phenotypes compared with non-invasive cancer cells [88] and results in increased IL-6 levels. Moreover S1P_1_ knockdown reduces IL-6/STAT3 signaling; this representing a pathway by which SK1/S1P regulates invasion [88]. HDL-S1P also promotes phosphorylation of STAT3 (at S727) and cell migration and this is reduced by S1P_1/3_ and S1P_2_ antagonists [89]. A link between S1P and STAT3 signaling is exemplified by studies showing that S1P enhances colitis associated cancer via a malicious amplification loop involving SK1, S1P_1_, NF-κB, STAT3 and IL-6 [90,91].

## 8. Clinical Evidence for Sphingosine 1-Phosphate Receptor 1 Modulators

There is intense interest in developing S1P receptor selective agonists/antagonist, some of which are in clinical trials [92]. For instance, ozanimod (RPC-1063, S1P_1/5_ modulator), reduces inflammation and disease parameters in multiple sclerosis and colitis rodent models [93]. A phase 2 trial of ozanimod in patients with relapsing multiple sclerosis showed reduced gadolinium-enhancing (GdE) Magnetic Ressonance Imaging (MRI) lesions [94]. In a preliminary trial, ozanimod induced a slightly higher rate of clinical remission among patients with moderate to severe ulcerative colitis (UC) compared with placebo [95]. Ozanimod is also currently being tested worldwide as an oral therapeutic in phase II clinical trials of ulcerative colitis [96]. In addition, 50% of psoriasis patients treated with the selective S1P_1/3/5_ modulator, ponesimod exhibited a 75% improvement in Psoriasis Severity Index (PASI) at week 16, whereas only 13.4% of the placebo group improved [97]. Ceralifimod (ONO-4641, S1P_1/5_ modulator) reduced the number of new/enlarging T2 lesions in multiple sclerosis patients switching from placebo to active treatment in the extension study (Drug Research and EvaluAtion in Multiple Sclerosis, DreaMS), while efficacy was sustained for patients on continuous active treatment [98]. Amiselimod (S1P_1_ modulator) improved MRI outcomes among patients with relapsing-remitting multiple sclerosis [99]. W-061, a prototype of ONO-464, improved Detran Sulphate Sodium (DSS)-induced colitis and significantly reduced the number of CD4^+^ T cells in the colon. Th17 and Th1 cells numbers were also reduced by W-061 treatment [100]. KRP-203, (2-amino-2-(4-3-(benzyloxy)phenyl)thio)-2-chlorophenethyl)propane-1,3-diol) an S1P_1/4/5_ modulator, alleviated chronic intestinal inflammation in IL-10 knockout mice [101]. Phase II clinical trials with ponesimod in multiple sclerosis demonstrated reduced GdE lesions [102].

## 9. Conclusions

There is significant translational activity and potential in developing novel S1P_1_ modulators. However, more subtle approaches appear available based on increased knowledge of S1P_1_ signaling and its regulation. For instance, the existence of different pools of S1P_1_ bound with accessory proteins or co-receptors raises the possibility of targeting these distinct S1P_1_ receptor pools for therapeutics. Examples could include the use of modulators to manipulate S1P_1_-β-arrestin-G-protein complex, S1P_1_-Receptor Tyrosine Kinase complex and HDL-S1P signaling in pathogenic conditions such as auto-immune disease, cardiovascular disease and cancer. In this regard, the use of in vivo reporters of S1P_1_ signaling seems poised to facilitate development of next generation small molecules and biologics that can be employed to therapeutically intervene in disease.
